# Technical Considerations in Remote LIMS Access via the World Wide Web

**DOI:** 10.1155/JAMMC.2005.217

**Published:** 2005

**Authors:** William Ulma, David M. Schlabach

**Affiliations:** 1 Accelerated Technology Laboratories Inc 496 Holly Grove School Road West End NC 27376 USA

## Abstract

The increased dependency on the World Wide Web by both laboratories and their customers has led LIMS developers to take advantage of thin-client web applications that provide both remote data entry and manipulation, along with remote reporting functionality. Use of an LIMS through a web browser allows a person to interact with a distant application, providing both remote administration and real-time analytical result delivery from virtually anywhere in the world. While there are many benefits of web-based LIMS applications, some concern must be given to these new methods of system architecture before justifying them as a suitable replacement for their traditional client-server systems. Developers and consumers alike must consider the security aspects of introducing a wide area network capable system into a production environment, as well as the concerns of data integrity and usability.


Journal of Automated Methods & Management in Chemistry, **2005** (2005), no. 4, 217–222
© 2005 Hindawi Publishing Corporation

### Technical Considerations in Remote LIMS Access via the World Wide Web

William Ulma and David M. Schlabach

*Accelerated Technology Laboratories, Inc, 496 Holly Grove School Road, West End, NC 27376, USA*

*Received 11 July 2005; Accepted 19 July 2005*

The increased dependency on the World Wide Web by both laboratories and their customers has led LIMS developers to take advantage of thin-client web applications that provide both remote data entry and manipulation, along with remote reporting functionality. Use of an LIMS through a web browser allows a person to interact with a distant application, providing both remote administration and real-time analytical result delivery from virtually anywhere in the world. While there are many benefits of web-based LIMS applications, some concern must be given to these new methods of system architecture before justifying them as a suitable replacement for their traditional client-server systems. Developers and consumers alike must consider the security aspects of introducing a wide area network capable system into a production environment, as well as the concerns of data integrity and usability.

### 1. INTRODUCTION

Remote access CRM solutions are becoming commonplace in the world of information technology. Laboratories that utilize an LIMS to control their information have the upper hand when making the decision to implement a front end for customers to access and submit data to the laboratory over the Internet. The benefits of CRM solutions can be obvious to those who desire lowered production costs, increased efficiency, and a reduction in customer service responsibilities. A remote access application can give a laboratory an advantage over its competitors by reducing the costs for the production and delivery of paper reports, and by allowing the delivery of analytical results and order statuses on a timelier basis.

When making the decision to implement a system, such as the one described above, there are a variety of details that need to be examined. These technical considerations range from the requirements of hardware and software resources to security and usability. We will attempt to investigate the components and practices that will allow laboratory managers and administration to successfully market and deploy a resource to their customers that will ultimately prove to be indispensable.

This document will attempt to inform and familiarize laboratory managers, IT managers, system administrators, and application developers to the practices and requirements central to deploying an auspicious solution to communicate information using the World wide Web. The topics covered are listed below and in the order of their importance:

- (i) network security and vulnerability,
- (ii) concurrency handling and data integrity,
- (iii) server resources and cost effectiveness,
- (iv) distribution and compatibility,
- (v) usability.

### 2. NETWORK SECURITY AND VULNERABILITY

The basic fundamentals of client/server architecture can be thought of as a system where transactions are occurring over a local area network (LAN), or in a more modern sense, over a wide area network (WAN) such as the Internet or a corporate Intranet. A remote data access system usually raises the issue of network and resource security. Most institutions invest a great deal of time and money in securing their internal networks to be safe from unauthorized intrusion, malicious software, and other exploitations that could disrupt day-to-day operations. Some of the biggest fears include file or data loss, network access issues, and even theft of resources. With proper setup and configuration practices even the most basic networks can prove invulnerable to becoming compromised.

The first step in securing your internal resources from the outside world is through the use of a router or hardware/software firewall. Firewalls function in a networked environment to prevent some communications forbidden by security policies. Through proper configuration of these devices network administrators can manage the transmission of data in and out of your local area network (LAN) and will

Correspondence and reprint requests to William Ulma, Accelerated Technology Laboratories, Inc, 496 Holly Grove School Road, West End, NC 27376 USA; E-mail: bulma@atlab.com.

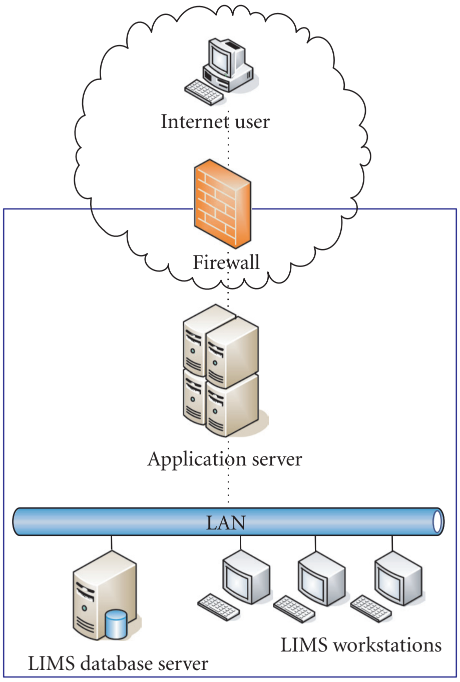

Figure 1: A network configuration example where network transmissions are controlled by a firewall and the database server cannot be accessed directly from the Internet.

also have the ability to monitor transfers to and from the network. (See Figure 1.)

The physical setup of a network will also benefit protecting data and resources. The implementation of a remote system does not necessarily mean that existing database and file servers must be accessible to the world. An IP address is a unique number, akin to a telephone number, used by machines (usually computers) to refer to each other when sending information through the Internet using the Internet protocol. When a computer system does not have a "live" IP address, one that is unknown outside of the LAN, direct connections cannot be made from machines outside of the LAN. By configuring the application server to access resources on the LAN or Intranet, it acts as a point of contact and becomes a conduit to database and file servers.

When data transmission security is an issue, the best option is to use secure socket layers (SSL) over your TCP/IP connection. SSL is a protocol that transmits your communications over the Internet in an encrypted form. SSL ensures that the information is sent, unchanged, only to the server you intended to send it to. Online shopping sites frequently use SSL technology to safeguard your credit card information, and health care sites are required to use SLL when transmitting medical information over the Internet. SSL sessions always begin with an exchange of messages called the SSL handshake. (See Figure 2.) The handshake allows the server to authenticate itself to the client by using public key techniques, and then allows the client and the server to cooperate in the creation of symmetric keys used for rapid encryption, decryption, and tamper detection during the session that follows. Transmitting large amounts of data using SSL can quickly cripple a webserver's performance because of math-intensive encryption and decryption calculations, when this becomes an issue, SSL accelerator appliances should be considered.

### 3. CONCURRENCY HANDLING & DATA INTEGRITY

Modern enterprise applications have one thing in common, multiple users accessing the system at the same time. In this type of scenario, you are bound to have users attempting to edit the same data at the same time, especially when there is a single data source associated with the application. The competition for data is known as a concurrency condition and can result in a lack of integrity in your database or worse, a loss of data.

The intended scope of a remote application will establish the need to handle data concurrency issues that may arise. If a system is intended to be primarily used as a reporting mechanism, then data concurrency will not be of much concern. When an application is taken to the next level and users will be interacting with the system on a multifaceted level, it is desirous to prevent users from modifying data in a way that affects other users.

The only way to prevent concurrency errors is to lock the records that are being edited. There are two basic approaches towards locking—optimistic and pessimistic (Figures 3 and 4). One would consider an optimistic locking

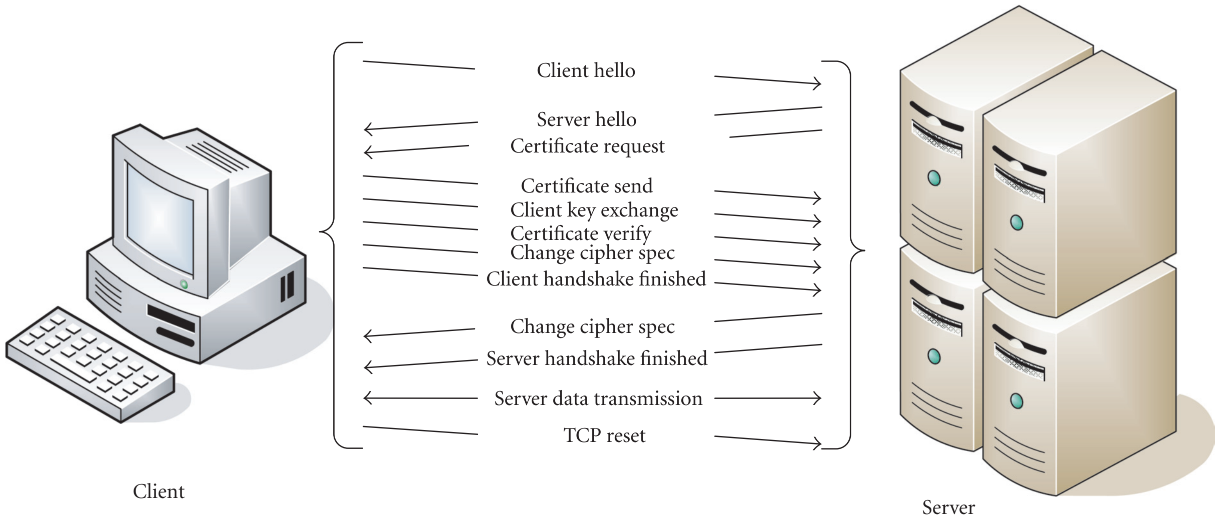

Figure 2: SSL handshake process to instantiate secure data transmission.

scenario when the likelihood of a concurrency condition is low. This is usually the case in systems where the activity is primarily additive, such as an order entry system. On the other hand, one would consider pessimistic locking when the likelihood of a concurrency condition is high. This is usually true of administrative or workflow-oriented systems.

Because this document focuses on remote LIMS access for CRM usage, rather than remote LIMS management, we will concentrate on optimistic record locking. Optimistic record locking is more practical in a detached database setting because it ensures the accessibility of information by not restricting access after a primary data request. Pessimistic locking requires that you remain connected to the database for the entire process, which is somewhat contrary to the .NET model.

Optimistic concurrency does not lock a row when reading it. When a user wants to update arow, the application must determine whether another user has changed the row since it was read. Optimistic concurrency is generally used in environments with a low contention for data. This improves performance as locking of records is not necessary, and locking of records requires added server resources. Also, in order to maintain record locks, a persistent connection to the database server is required. Because this is not the case in an optimistic concurrency model, connections to the server are free to serve a larger number of clients in less time.

In an optimistic concurrency model, a violation is raised if, after a user receives a value from the database, another user modifies the value before the first user has attempted to modify it. Today's web technology development platforms such as the .NET framework utilize optimistic record locking as the default method.

### 4. SERVER RESOURCES AND COST EFFECTIVENESS

Today's computers are very powerful in comparison to the hardware available only a few years back. These enhancements allow network and enterprise servers to handle hundreds, if not thousands, of simultaneous data requests and processing instructions without any degradation in performance. The core elements that will retain high-performance operations are the selection of a proper client/server model, database connectivity mechanism, and lightweight record locking methods. The desired choices for these three essential items are a thin client system with a disconnected database model using optimistic record locking. Though a thin client system handles the bulk of the data processing operations on the server, strict control of the database connectivity and good record locking techniques will make the best of high speed data availability.

The majority of today's remote applications are built around the thin client system model which one source simply defines as a client designed to be especially small so that the bulk of the data processing occurs on the server. A more detailed explanation is acknowledged as an architecture that retains user interface software on a client machine, but moves business logic (rule) software to a central server platform called an application server device. While the application server may require more computing resources due to the additional processing load that it must support, there is an even greater reduction in the amount of computing resources required (in the aggregate) on the client machines. Thin clients are becoming the preferred model for remote applications that are intended to be used as customer relationship management solutions because they prove to be less costly in the long run, and require less commitment, for both the administrators and the end user. (For a comparison between fat and thin clients see Figure 5.)

Before employing a thin client, certain steps must be taken to preserve resources on the application server, since it will be handling all of the data processing and operation requests from the client. The system must be architected in such a way that it does not leave any residual connections or locks on information that would result in decreased system functionality.

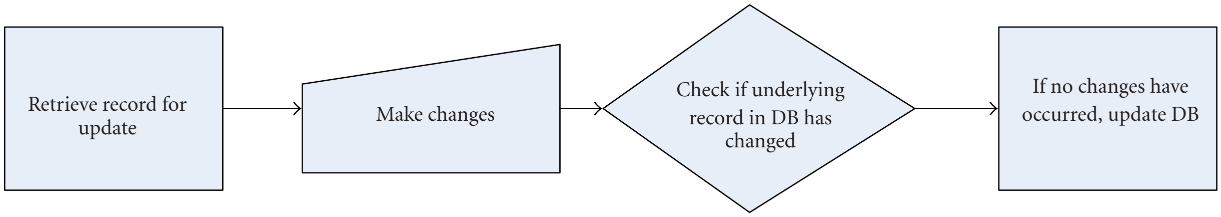

Figure 3: Optimistic record locking scenario.

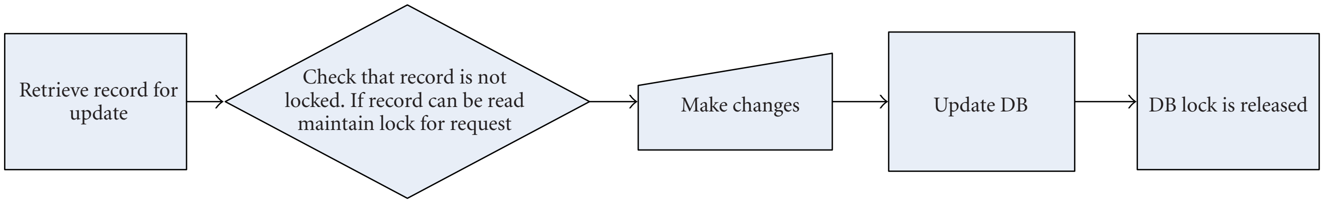

Figure 4: Pessimistic record locking scenario.

The use of disconnected database access is the foremost step towards increasing the efficiency of the server's performance. In previous versions of database access technologies, it usually was a continuously connected client. In such a model, an application creates a connection to a database and keeps it open for the life of the application or at least for the amount of time that data is required. Today's database development technologies, such as ADO.NET, address these issues by implementing a disconnected database access model by default. In this model, data connections are established and only left open long enough to perform the requested action.

Resource preservation can also be accomplished through the use of caching methods whereby dynamic pages are delivered as static pages to minimize data access operations. For example pages or data that are infrequently updated, such as system notifications, can be stored as cached information for faster loading. This frees the server for more data intensive processes while eliminating HTML generation and reducing page delivery time.

### 5. DISTRIBUTION AND COMPATIBILITY

One obstacle, usually overlooked, when implementing a remote system is the means for distributing the front-end client to the users. If the client is developed as a standard or fat client system, then administrators must determine a method to dispense and maintain the client application, and if the fat client is operating system (OS) proprietary it may not be compatible.

An HTML front end through a web browser is the best option when havingto take cross-platform compatibility into consideration. While this document considers development of the back end to take place on a Windows/.NET application server, clients may tend to rely on a variety of operating system (OS) platforms. UNIX, Linux, and the Macintosh OS are some examples where distributing a Win32 thin client application would limit the extent of client distribution. (See Figure 6.)

Maintenance of a web-based application is also eased because the client uses the most updated versions of pages directly from the server without the need to install any software or reconfigure settings and preferences.

In addition, it is far easier to distribute application software changes from a centralized application server than to physically distribute that software to client workstations using fat client architecture. As a result, software updates can be made available on a timelier basis and at significantly lower cost using thin client architecture. Because the lifetime of HTML interfaces ends when a user closes their web browser, administrators can be sure that all clients are interfacing with the back end with the latest versions by simply updating files on the application server.

### 6. USABILITY

A concern that may deter customers from taking advantage of remote access tools provided to them is usability. Most people deal with one or more pieces of productivity software on a day-to-day basis to get their work done, and if a tool doesn't integrate well into their current practices, then it may go unused. When designing the workflow of the remote application, keep in mind that the system must not only be easy to use, with powerful features, but it must also be desirable.

Familiarity is crucial when designing the interface, by keeping in mind the applications and processes that your customer is habituated to, you can raise the level of appeal that your system communicates. Today's users are accustomed to user controls found in common office applications, items such as buttons, icons, combo boxes, and tree views. These interface types are known as WIMPs (windows, icons, menus, and pointing devices) and are found in all of today's operating systems desktop environments.

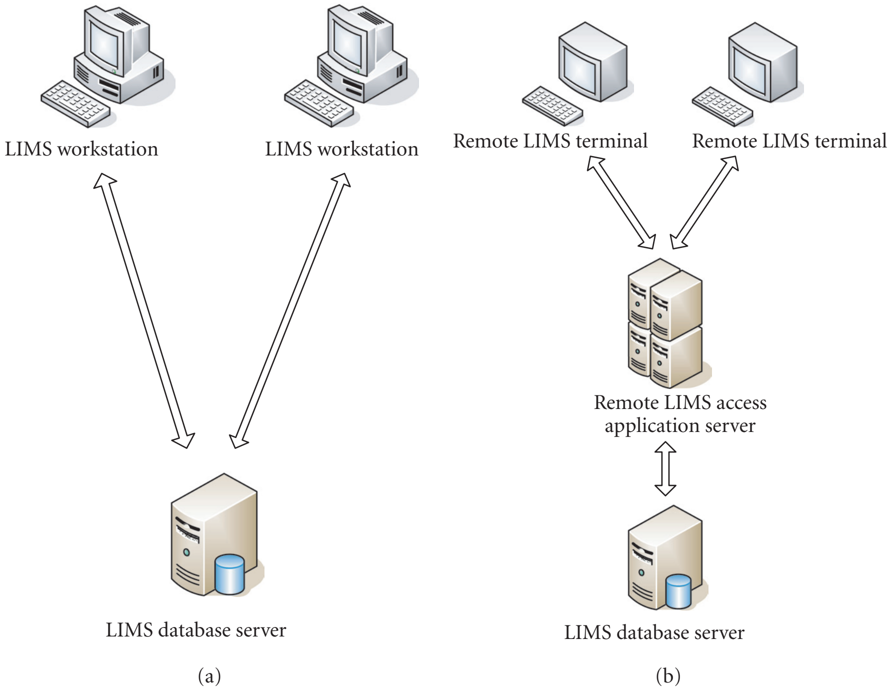

FIGURE 5: Fat client (a) versus thin client (b) system models.

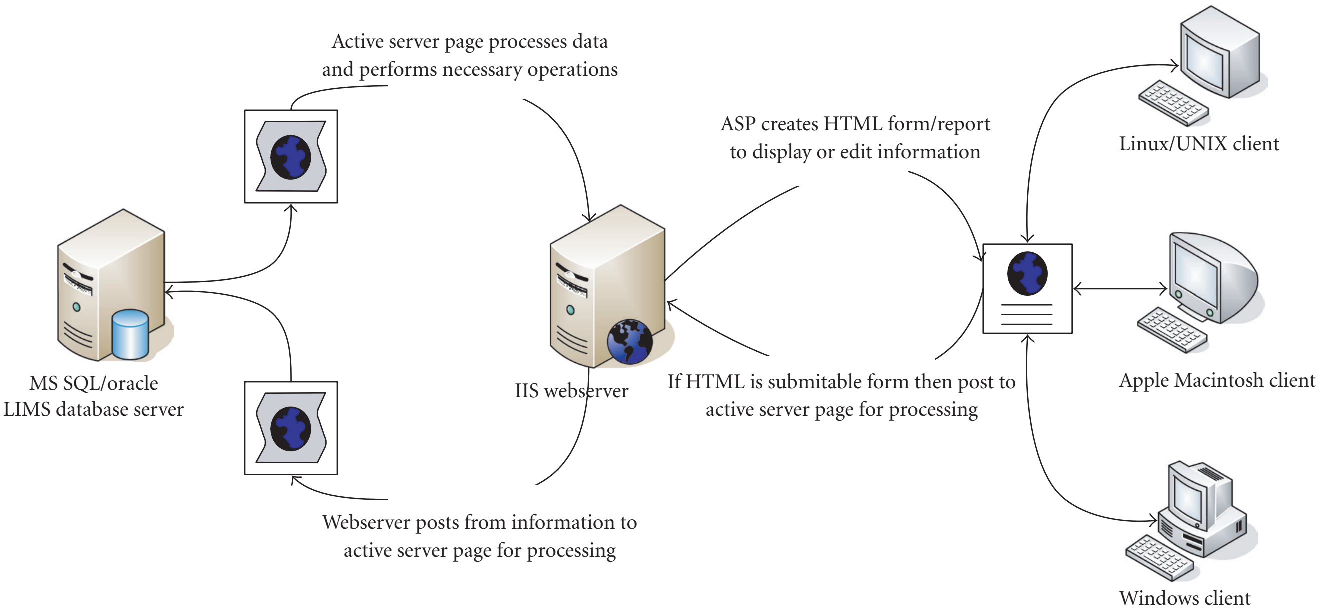

FIGURE 6: Server side scripting to HTML for cross platform compatibility.

Creating a radically styled interface will not only lead to increased training time, which may once again increase the level of costs and responsibility required by both the administrator and the end users. As such the advent of DHTML gives developers the ability to create feature rich environments that do not overwhelm the end user. By combining the abilities of standard HTML and DHTML, along with other technologies such as XML, the end user will adapt easily to the system and its functionality will incorporate into their existing procedures. A user interface must be designed with fluency in mind and the success of the tool will be ultimately proven not only by how well it works, but by how often it is used.

Another concern that must be looked at carefully when in view of usability for a remote LIMS system is limiting the number of data requests that are made to the server. Limiting data requests not only benefits the preservation of server resources but also reduces frustration when attempting to work with the application. Most data-driven systems use the same fundamental design for selecting data, which is based around the concept of a connected database model. For example, a user selects an item from a drop-down list and the next drop-down list or combo box is populated based on data from the initial selection.

This workflow design is effective; however each time a change is made we are opening and closing a connection to the server. That is fine when transmitting over 100 or even 10 Mbps local area network (LAN) connections, however once you reduce that to broadband access over a WAN and consider routing jumps alongside server side page generations, dropdown updates can be reduced to a crawl. Deciding which data elements a user needs to complete an operation without having to make frequent requests to a server will benefit users who utilize low-bandwidth connections while decreasing network transfer to, and processing time on, the application server.

### REFERENCES

[1] Webopedia: The only online dictionary and search engine you need for computer and Internet technology definitions, http://www.webopedia.com.

[2] Microsoft Developers Network (MSDN) Library, http://msdn.microsoft.com/library.

[3] Zeus Technology, http://www.zeus.com/products/zws/security/ssl.html.

[4] 15 Seconds : Handling Concurrency Issues in .NET, http://www.15seconds.com/issue/030604.htm.

[5] Electronic Cottage Associates - Glossary, http://www.eleccott.com/glossary.htm.

[6] useit.com: usable information technology, http://www.useit.com/.

[7] ASP 101-Security Features in ASP.NET-Authentication, http://www.asp101.com/articles/cynthia/authentication/default.asp.

